# Supplementary material for: The hierarchical packing of euchromatin domains can be described as multiplicative cascades
Source: PLoS Comput Biol. 2021 May 5;17(5):e1008974. doi: 10.1371/journal.pcbi.1008974 (PMC8128263; doi:10.1371/journal.pcbi.1008974)
Supplement: Attachment — Submitted filename: ResponseLetter_3April2021.pdf [file pcbi.1008974.s005.pdf]

Author response letter, PLOS Computational Biology (PCOMPBIOL-D-20-02010) - [EMID:6e41c06170251959]

To the editors and referees, dear Dr. Ioshikhes, Dr. Noble, and Dr. Hildebrand,

We very much appreciate your positive overall assessment and all-around constructive requests.

We have addressed all requests, the responses to which you can find in our detailed reply below. In our opinion, the revisions have made for a much improved, more rigorous, and more accurately formulated manuscript. I hope your concerns regarding our study can thus be appropriately accounted for.

Further, we submit two versions of the updated manuscript, one with marked-up changes in red font as part of this response letter, and one clean version as a manuscript file in the submission system.

The manuscript has been prepared in Overleaf. In the case of a positive assessment of our resubmission, we can provide the LaTeX source to facilitate editorial processing.

With kind regards,

Lennart Hilbert

Reviewer #1: The article provides an interesting application of a theoretical concept of multiplicative cascades to describe the processes governing euchromatin packing. This theoretical framework has the potential to be used to test mechanistic models of euchromatin organization.

Major revisions:

The article needs to go into further detail of what the descriptive parameters P1, P2, P3, P4 mean in the context of generating the multiplicative cascade images. There is needs to be additional methodological discussion of why the decrease of the probability values from P1 to P4 is common to all patterns of euchromatin observed in the microscopy images.

We should have explained the ordering of P1 to P4 better. The four probabilities are randomly drawn at first, but then ordered from largest to smallest before further analysis.

We now added this explanation to the Results section:
"To allow the analysis of parameter distributions, we ordered P_1 to P_4 in descending order throughout. This order does not affect the partitioning process where P_1 to P_4 are randomly assigned to the four sub-squares."
and, referring to the decrease from P_1 to P that is seen throughout:
"Note that this decrease is a result of the sorting of the probability values in descending order. Based on the fitting to representative microscopy images, however, this decrease of the parameter values seems to be the more pronounced the more compacted the euchromatin is in the target images (Fig. 4F)."

We also added this explanation to the caption of Figure 4:
"The values P_1 to P_4 were sorted in descending order before plotting. Note that this sorting did not affect the image generation."

We also tried to explain this better in the Methods section:
"The four probabilities are always sorted in descending order ($1 \geq P_1 \geq P_2 \geq P_3 \geq P_4 \geq 0$). Note that this sorting does not affect the cascade process."

The authors discuss testing mechanistic models for euchromatin organization such as microemulsification, however they do not provide any direct examples of how multiplicative cascades can be applied to test them. Since the authors already utilize microemulsion theory fitting functions in their methodology, it would be extremely valuable for the readers to see the utility of multiplicative cascades as a test in verifying this model.

We now applied the cascade fitting approach on microemulsion simulation results from our recent paper (Hilbert et al. 2021). While the results do not look completely identical, we indeed find the same qualitative relationship between the parameters P1 to P4 and image contrast, in the microscopy data and in the microemulsion simulations.

We added the following text and an according SI Figure (new Fig. S4) to the Results section:
"This conclusion was also supported by an application of the same cascade fitting approach to simulations of euchromatin microphase separation (Fig. S4) obtained from a lattice model of microemulsification (Hilbert et al. 2021)."

The process for generating concentration profiles by multiplicative cascades is done through randomly assigning one of the 4 parameters to each pixel. It would be useful to mention how

rerunning the concentration profiles using the same set of parameters affects the resulting images, particularly in the context of their ability to reproduce euchromatin patterns as seen in Figure 4.

To assess in how far the stochastic image generation by cascades interferes with the correct estimation of model parameters, we applied our fitting procedure to images that were themselves generated by cascades with prespecified parameters (new Fig. S2). We find that two refitting approaches can reliably capture the original parameters: picking the top cascade as well as the top-100 from an ensemble of 10,000 candidate cascades. We thus conclude that stochastic effects from rerunning cascades do not interfere with the fitting approach to any obvious extent. We added the following sentence to the Results section:
"We verified that this fitting approach can accurately recover known probabilities (Fig. S2), so that we can use it for parameter estimation on a larger scale."

Additional comments:

The article should be carefully reviewed for typos. Specifically, in the methods on page 13, (0 ≤ P1, P2, P3, P4 ≤ 1) appears to be the reverse of the decrease of the probability values as described in the results and figures.

We checked again for grammar and spelling mistakes.

We fixed the point on P_1 to P_4, thank you for pointing it out.

The word organzation is misspelled in the discussion on page 13.

Fixed, thank you

Reviewer #2: Summary:

This paper presents a new method to model super-resolution microscopy of euchromatin folding within the nucleus, based on a theory of hierarchical chromatin folding using multiplicative cascades. Using this method, the authors determine specific values of 4 parameters required for the multiplicative cascades that can be applied to mimic microphase separation observed in images of euchromatin, which may be useful for testing whether other types of theoretical models accurately reproduce chromatin folding, or for comparisons to future experimental data. In addition, this paper presents an optimized DNA stain/mounting media combination for STED microscopy of nuclear structure in zebrafish embryos. The computational method as applied to chromosome folding super-resolution imaging is original, innovative, and is of importance to researchers in the field of chromosome folding, as it may facilitate comparisons between imaging data and modeling data generated by other means by using the identified multiplicative cascade parameters as a measure of similarity between real and modeled data.

However, there are some conclusions that require additional analysis to be fully supported. In addition, some parts of the methodology are not adequately described or explained, and the underlying computational analyses, raw imaging data and modeling results do not seem to have been made available, and should be included in the final publication.

Major Concerns:

On Page 7, the authors state that "euchromatin intensity distributions showed properties typical of

multi-fractals rather than conventional fractal sets", however Supplementary Material Figure 5 includes only the multi-fractal analysis on the chromatin images, without controls to support this statement. This point would be better supported by also including both a conventional fractal analysis showing that this is not supported by the data, and a comparison to a known multifractal dataset such as the clouds described in the example, or the referenced soil dataset (Adolfo N. D. Posadas, Daniel Gim ́enez, Roberto Quiroz, and Richard Protz. Multifractal Characterization of Soil Pore Systems. Soil Science Society of America Journal, 67(5):1361–1369, 2003.).

We now included in SI Fig. 5 (now called Fig. S1) three additional reference cascades. These cascades are based on probability values from literature, which lead to the generation of one mono-fractal and two multi-fractals (Martinez et al.). These added images clearly show the difference between mono-fractals (single point in the f-alpha spectrum, and a constant fractal dimension) and multi-fractals (extended f-alpha spectrum, varying fractal dimension).

To explain our argument better, we now included the following text in the Results section:
"Multi-fractals are commonly characterized using a multi-fractal spectrum, also called an f−α curve (for details, see Methods and Materials). Multi-fractal spectra obtained from our microscopy data (Fig. S1) were similar to those seen for other multi-fractal systems (Posadas et al., Meneveau et al.). This similarity implies that euchromatin forms patterns with multi-fractal properties."

On Page 7, the authors state "Image contrast and correlation length values of these examples were also comparable to the values obtained for the smooth, intermediate, and coarse patterns in our microscopy data", However, in Figure 3B the Lcorr values shown for modeled images that are visually similar to the real images have a much wider range than the Lcorr from the corresponding real images. It would be useful to also show the images corresponding to extremes of Lcorr and Cdna from the model, and representative images that more closely match both the Lcorr and Cdna values. In addition, the authors should add a description of the criteria that were used to choose these examples – were they chosen visually to be similar to the images in Figure 2, by Lcorr and Cdna value, or by Mass Distribution and Radial Correlation similarity as in figure 4?

We followed this suggestion for changes to Fig. 3. We added two more examples of cascade-generated images, which are close to the C_{DNA} and L_{corr} values of the experimental data (Fig. 3C). To explain this better, we also slightly rewrote the description related to this figure:
"Also, the image contrast and correlation length values of some of these examples were comparable to the values obtained for the smooth, intermediate, and coarse patterns in our microscopy data (Fig. 3C)."

The different patterns of euchromatin organization shown in Fig. 3B are chosen based on visual assessment and the C_{DNA} and L_{corr} values. This initial choice is done purely to illustrate the different types of patterns. In Fig. 3C we now removed the clear reference to the smooth, intermediate, and coarse pattern, to not give the impression that a quantitatively verified correspondence has been established at this point – this is only done thoroughly in Fig. 4.

In Figure 4 A and B, the top generated image does not look very similar to the top microscopy image, however the metrics used to compare them in the rest of the figure look similar. This suggests that some additional metric may be required to be able to robustly compare between real and modeled data, as visually these two images look quite different, and calls into question the later analysis of cascade parameters across all nuclei, as it is not clear how well the modeled images are matched to the real images. A more comprehensive analysis of the matching of modeled and real data would improve this point.

The visual discrepancy between the images stems from omitting detector noise from the image generation process. In this situation, small fluctuations in concentration are amplified in the display with an adjusting color map, as we use here. We now added this simulated detector noise (see below for details). We updated parts of Fig. 3, the entire Fig. 4 and SI Fig. 7 (now called Fig. S3) accordingly. As a result, the images in Fig. 4B look more similar to those in Fig. 4A now.

In addition, we prepared a new Fig. S2, where we assess how the known parameters of model cascades are recaptured by our fitting approach. For all patterns, there is a clear connection between the pre-specified and the recovered parameters. In almost all cases, this looks extremely close to the original values. Only for the most unstructured pattern, a slight deviation is seen for one of the two fitting approaches we test, and even in this case the recovered parameters are close. This level of performance looks sufficient, in our opinion.

The reliability of our fitting approach is also supported by a third line of argument. In the new Fig. S4, we demonstrate that the same relationship between cascade parameters P1 to P4 and image contrast is obtained by fitting to microemulsion simulation results instead of microscopy data (see also reply to referee #1).

On Page 9, the authors state that “For all euchromatin patterns, a gradual decrease of the probability values starting from P1 via P2 and P3 towards P4 can be seen”, however, it is unclear how P1 to P4 are ordered – are they sorted randomly/based on which was picked first-fourth, or are they sorted based on decreasing value, in which case this would be the only possible outcome? This should be clarified in the methods. In addition, if the ordering is important, are there any examples of images where the values increase or have no trend from P1 to P4, and how do these images look compared to real data? The conclusion that there is more of a difference between the probabilities in more compact states is supported, but I am not sure that the order itself matters based on these results.

We should have explained the ordering of P1 to P4 better. The four probabilities are randomly drawn at first, but then always ordered from largest to smallest before further analysis. As the referee states, the point is not the decreasing order as such, but rather that the decrease is more pronounced for more compact states (Fig. 4 and Fig. S3).

We now added this explanation to the Results section:
“For the analysis of parameter distributions, we ordered P_1 to P_4 in descending order throughout. This order does not affect the partitioning process where P_1 to P_4 are randomly assigned to the four sub-squares.”
and, referring to the decrease from P_1 to P that is seen throughout:
“Note that this decrease is a result of the sorting of the probability values in descending order. Based on the fitting to representative microscopy images, however, this decrease of the parameter values seems to be the more pronounced the more compacted the euchromatin is in the target images (Fig. 4F).”

We also added this explanation to the caption of Figure 4:
“The values P_1 to P_4 were sorted in descending order before plotting. Note that this sorting did not affect the image generation.”

We also tried to explain this better in the Methods section (see also reply to referee #1):
“The four probabilities are always sorted in descending order ($1 \geq P_1 \geq P_2 \geq P_3 \geq P_4 \geq 0$). Note that this sorting does not affect the cascade process.”

A simple case of a cascade with non-decreasing values for P1, P2, and P3 can be seen in the new Figure S1, for the case of a mono-fractal (1,1,1,0).

On Page 9, the authors state that “Now that we established multiplicative cascades as a process that can generate domain-within-domain patterns, we would like to know how the different patterns of euchromatin organization are represented by cascades with specific properties”. How has the domain-within-domain pattern itself been tested? This is not clear from the text.

Upon this comment and the one below on the title, we changed the title to “The hierarchical packing of euchromatin domains can be described as multiplicative cascades”. The reason is that, unintentionally, the title gives the idea that one fundamental domain of euchromatin is placed within another fundamental domain. Rather, we wanted to express that several fundamental domains associate into “super-domains”, which again can associate into even bigger “super-domains”. With a couple of months, looking at the title again, we agree that it was not well-chosen, and hope the new title fits better. We changed the wording in abstract, author summary, introduction, results, and discussion accordingly. Changes are marked in red.

On Pages 9 and 11, the finite scale of the microscope vs. the scale-free characteristic of multiplicative cascades is discussed, however it should be noted that in addition to the finite scale of the microscope, the chromatin is also constrained by the size of nucleosomes and other types of DNA binding proteins, and even the persistence length of DNA, and cannot in reality remain scale-free at very small distances, even if the microscopy was not resolution limited. This may affect the conclusions about the applicability of the multiplicative cascade to studying chromatin structure at certain size ranges.

This is an important point. We added the following section to our Discussion:
“Based on previous work, two limits to a truly scale-free packing can already be identified. First, the scaling behavior changes distinctly at the transition from large-scale chromatin organization to the packing into contact domains (Rao et al. 2014, Huang et al. 2020). This transition occurs at the scale of a few megabases of sequence length, and at three-dimensional domain sizes of approximately 100 nm (Kempfer et al. 2020, Mateo et al. 2019, Szabo et al. 2020, Bintu et al. 2018, Cardozo Gizzi et al. 2019, Szabo et al. 2018, Miron et al. 2020). Second, going to yet smaller scales of organization, the structure of the chromatin fiber itself is revealed at sequence lengths of a few 100 base pairs, and distances in three-dimensional space of 10 to 30 nm (Ricci et al. 2015, Ou et al. 2017, Kempfer et al. 2020}. The chromatin fiber as the fundamental level of genome organization thus represents a hard lower limit on any scale-free organization. The resolution achieved by STED microscopy of DNA in cell nuclei, in practice, ranges from 40 to 100 nm, thus not facing this limit.

In Figures 2 and 3: It would be useful to add an explanation of why the F(k) fits and data diverge above 10^1.2 for the real data but not for the modeled images. The model generated images seem to be close to the F(k) fit for a much broader range than the real data – is this expected, and how does this affect the results? How is this related to the result in Figure 4 comparing the blurred vs unblurred simulated images?

Following the referee’s comment, we found that addition of simulated detector noise to the cascade-generated images reproduces the deviation at high values of k. We extended Fig. 3 to demonstrate this effect, so that we can now pinpoint the influence both of the limited resolution and detector noise. We took this modification of the image generation process forward and also corrected Fig. 4 and SI Fig. 7 (now called Fig. S3) accordingly. The addition of noise also addresses this referee’s

concern with one simulated image in Fig. 4 that looked different from the original microscopy data that it approximates.

In the Method section, we modified the description of the image generation process accordingly:
"To normalize the concentration profiles, first all pixels were divided by the mean concentration. Then, a pixel size closely matching the pixel size in the microscopy data was assigned (30 nm unless stated otherwise). Generated microscopy images were obtained by applying a Gaussian blur that approximates the limited resolution of the microscopy images (kernel width specified as σ_1). Noise in the photon detector of the microscope was approximated as a Poisson process by adding exponentially distributed random numbers to every pixel (emission rate specified as λ). The image was then subjected to another Gaussian blur filter (kernel width σ_2), as was done for microscopy images. To remove background intensity similar to microscopy images, the 0.01 percentile intensity value was subtracted from all pixels."

Minor Comments:

For Figure 2, please add a description of the method that was used to classify the nuclei into interphase vs. cell division, and into the different patterns of euchromatin organization.

We remove nuclei before, during, and shortly after cell division based on visual assessment. We chose to use a visual assessment, because in a data set of less than 100 nuclei also an automated approach would, by virtue of parameter adjustment, effectively perform with as much choice how to include or exclude nuclei. To explain the removal of nuclei, we now added this to the figure legend:
"Examples of nuclei before, during, and after cell division indicated as dashed boxes – nuclei in these cell cycle stages were removed based on visual assessment."

The different patterns of euchromatin organization shown in Fig. 2B (and also Fig. 4A) are chosen based on visual assessment and the C_{DNA} and L_{corr} values. This initial choice is done purely to illustrate the different types of patterns. Given that the euchromatin organization displays a continuum, as seen in Fig. 2C, we then proceed to analyze all nuclei without sorting into distinct classes. This analysis without classification can be seen in Fig. S3. To more immediately provide the idea of a continuum of configurations, we now modified the according section in the Results as follows:
"After excluding nuclei of cells preparing for, undergoing, or exiting division, we found a continuum of organization patterns. To illustrate this continuum, we display three typical patterns of organization, which we refer to as smooth, intermediate, and coarse (Fig. 2A)."

In Figure 3: As noted in the text on page 7, In C, Both Lcorr and CDNA for the model have a much wider range than the real data – please expand on the significance of this.

We now expanded on that point:
"Note, however, that the generated patterns spanned a distinctly wider range of values, suggesting that the observed types of euchromatin organization are contained in a subset of all types of organization that can be generated by multiplicative cascades."

This wider range of patterns in the cascade generation process can also be seen in the additional examples shown in Fig. 3C (see above response to this referee).

In all figures, it would be useful to note what % of the nuclear area is included in each of these analyzed images, and to note whether multiple areas from one nucleus always show the same type

of structure, or can multiple types of structure be found in one nucleus? How does this compare for the modeled images?

Regarding the fraction of the nucleus that was used in analysis, we added the following explanations to the Materials and Methods section:
Microscopy: "For full 3D stacks, the acquisition region was chosen to cover the entire nucleus. For 2D images that were used for C_{DNA} and L_{corr} calculation and for cascade parameter adjustment, a square region was acquired that fit within the nucleus without intersecting the nuclear envelope. The nuclear envelope was excluded to prevent previously observed edge effects (Hilbert et al. 2021)."
Image analysis: "For all analyses described below, square images from within a given nucleus were used, making segmentation unnecessary."
Seeing that in all cases where we analyze the euchromatin pattern, we fit a square into an approximately circular nucleus, a geometrical consideration would imply that we use approximately half of the total area of the section through the nucleus in the analysis.

The envelope does exhibit a different type of organization that does not primarily inform on euchromatin packing, and perturbs the analysis. We addressed this point in a previous publication (Hilbert et al. 2021) and would like to avoid repeating this in a second study.

In any case, we wanted to demonstrate for two example images that using only a part of a given nuclear image does result in quite different statistical representations of the data. We have obtained mass distributions, correlation functions, and structure factor curves from microscopy images after cutting them into four quadrants. The results are shown below for the referee, and indeed show strong differences between the quadrants. It is because of these fluctuations that stem from using subregions, that we adjust the cascades on the overall image, which is fitted into the inner square of the nucleus. We thus covering different regions as good as possible within the technical limitations.

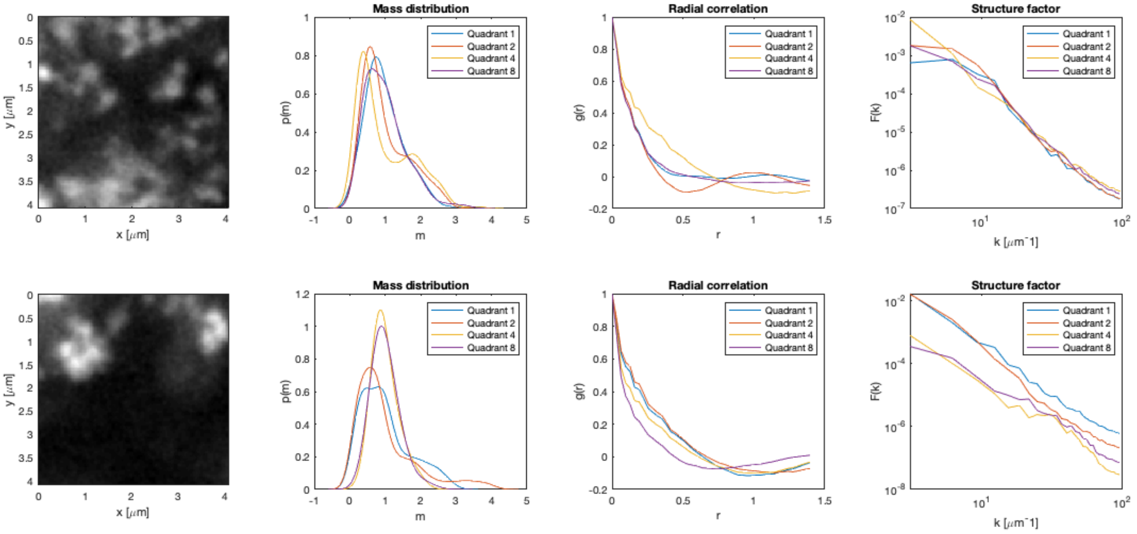

In case the question on "modeled images" refers to images resulting from our previous microemulsion model (Hilbert et al. 2021), we have now included a new Fig. S4 that shows that fitting the cascade parameters to simulated image data results in the same overall conclusion: a coarser euchromatin organization is captured by a steeper descent of the probability values used in the cascade-based image generation.

Additional protocols/algorithms that are not included, and would be beneficial to share: CellProfiler pipelines used for analysis, Matlab scripts used for analysis, software used for modeling images using multiplicative cascade.

We only used CellProfiler and MatLab in the analysis and generation of our images. We have assembled and documented all raw data and scripts used in the study, and prepared a ZIP file that contains all of it. We would upload this file in case of acceptance, or also at an earlier time if needed.

Original imaging data and results from modeling are not available. Something like https://www.ebi.ac.uk/bioimage-archive/ or https://figshare.com/ or https://idr.openmicroscopy.org/ might be appropriate.

See above, in case of acceptance we think the best way of release would be as supporting data. The data are ready to submit.

Descriptions of Lcorr calculation and the multi-fractal analysis method should be more detailed, some of the variables in the equation seem to be not defined or explained in the text of the methods.

Both parts of the Materials and Methods section were rewritten to better explain the approach and introduce the different quantities at hand.

Funder website URLs are not included in the financial disclosure statement, and role of funders in the study is not included in the financial disclosure statement.

We added the following statement to the Acknowledgments: "*The funders had no role in study* design*, data collection and analysis, decision to publish, or preparation of the manuscript."*

We also added all funding agency website URLs.

Include n for imaging experiment quantifications in Figure 1.

We added the following to the legend of Figure 1: "Number of assessed nuclei: SiR-DNA n= 5,10,5; SPY650-DNA n= 6,8,13; SPY595-DNA n= 7,13,12."

In the title, "The domain-within-domain packing of euchromatin can be described as multiplicative cascades", the "domain-within-domain" part is not fully supported by the current version of the paper.

That is a completely valid point, and we changed the title to "The hierarchical packing of euchromatin domains can be described as multiplicative cascades". For an explanation, see above comment.

Have all data underlying the figures and results presented in the manuscript been provided?
Large-scale datasets should be made available via a public repository as described in the *PLOS Computational Biology* data availability policy, and numerical data that underlies graphs or summary statistics should be provided in spreadsheet form as supporting information.

Reviewer #1: Yes

Reviewer #2: **No:** CellProfiler pipelines used for analysis, Matlab scripts used for analysis, software used for modeling images using multiplicative cascade, original imaging data and results from modeling should be made available.

All requested data are ready for upload, see above.
